# Modelling the influence of naturally acquired immunity from subclinical infection on outbreak dynamics and persistence of rabies in domestic dogs

**DOI:** 10.1371/journal.pntd.0009581

**Published:** 2021-07-20

**Authors:** Susannah Gold, Christl A. Donnelly, Rosie Woodroffe, Pierre Nouvellet

**Affiliations:** 1 Institute of Zoology, Zoological Society of London, London, United Kingdom; 2 MRC Centre for Global Infectious Disease Analysis, Department of Infectious Disease Epidemiology, School of Public Health, Imperial College London, London, United Kingdom; 3 Department of Statistics, University of Oxford, Oxfordshire, United Kingdom; 4 School of Life Sciences, University of Sussex, Falmer, Brighton, United Kingdom; University of Glasgow, UNITED KINGDOM

## Abstract

A number of mathematical models have been developed for canine rabies to explore dynamics and inform control strategies. A common assumption of these models is that naturally acquired immunity plays no role in rabies dynamics. However, empirical studies have detected rabies-specific antibodies in healthy, unvaccinated domestic dogs, potentially due to immunizing, non-lethal exposure. We developed a stochastic model for canine rabies, parameterised for Laikipia County, Kenya, to explore the implications of different scenarios for naturally acquired immunity to rabies in domestic dogs. Simulating these scenarios using a non-spatial model indicated that low levels of immunity can act to limit rabies incidence and prevent depletion of the domestic dog population, increasing the probability of disease persistence. However, incorporating spatial structure and human response to high rabies incidence allowed the virus to persist in the absence of immunity. While low levels of immunity therefore had limited influence under a more realistic approximation of rabies dynamics, high rates of exposure leading to immunizing non-lethal exposure were required to produce population-level seroprevalences comparable with those reported in empirical studies. False positives and/or spatial variation may contribute to high empirical seroprevalences. However, if high seroprevalences are related to high exposure rates, these findings support the need for high vaccination coverage to effectively control this disease.

## Author summary

Rabies-specific antibodies in unvaccinated domestic dogs have been reported in several studies, in some cases in a substantial proportion of the population. These antibodies may be the result of non-lethal rabies exposure leading to naturally acquired immunity, which could influence rabies transmission and persistence. In this study, a model was developed to consider a range of scenarios for naturally acquired immunity in a domestic dog population. Model outputs for rabies incidence and seroprevalence, the proportion of the population with rabies antibodies, were compared to estimates from empirical studies. Our results indicate that naturally acquired immunity could contribute to rabies persistence by limiting disease incidence and preventing population extinction. However, taking into account spatial structure and human response to high rabies incidence showed that rabies could persist without naturally acquired immunity, although immunity may contribute to the low incidence observed for endemic rabies. Assuming higher rates of subclinical exposure led to seroprevalences comparable to the estimates from empirical studies. However, uncertainty remains over the interpretation of rabies antibodies in unvaccinated individuals, and false positives may contribute to the high seroprevalences reported.

## Introduction

Rabies is a zoonotic disease, caused by a neurotropic virus in the lyssavirus family. Despite eradication having been achieved in some parts of the world, the disease still presents a significant public health burden, particularly in rural Africa and Asia [1]. All mammalian species are susceptible to rabies, however only a limited number, primarily bats and carnivores, are able to maintain the virus within their populations [2]. In Africa, domestic dogs are the primary host of rabies and cause the majority of human cases, therefore controlling the disease in this species is key to preventing human rabies deaths [3].

A number of models have been constructed for rabies dynamics in domestic dog populations [4,5]. One assumption commonly used in these models is that immunity only occurs through vaccination, and not as a result of non-lethal exposure (for example [4–7], but see [8]). While rabies is usually fatal following the appearance of symptoms, Hampson et al. (2009) estimated that 51% of bite exposures in domestic dogs did not lead to clinical infection [9]. Of these exposure incidents, it is unclear whether the virus always fails to establish, and the host remains susceptible, or whether in some case the virus is cleared by the host’s immune system, with subsequent development of protective immunity. While recovery from clinical rabies is rare, experimental studies have shown that non-lethal rabies exposure can occur, with exposed individuals showing no, or only minor, symptoms [10,11]. Under field conditions, there has been little consideration of seroconversion, the development of a specific antibody response, following rabies exposure. However, Cleaveland and Dye (1995) reported that of 17 dogs bitten by two suspected rabid individuals, 12 survived exposure of which four then seroconverted. As well as subclinical bite exposure, there is also limited evidence that oral exposure, for example from feeding on infected carcasses, can lead to development of rabies-specific antibodies in carnivores [12,13]. The potential for oral exposure to lead to development of rabies immunity is also supported by the success of vaccination campaigns using oral vaccines [14,15].

Rabies-specific antibodies have been detected in healthy, unvaccinated individuals across a number of domestic dog populations in rabies endemic areas, with a wide range of seroprevalences (the percentage of the population with detectable rabies-specific antibodies) reported [16]. There are a number of challenges to interpreting serology, including that different studies have used different tests and cut-offs to define seropositives [16,17]. However, given that clinical rabies typically occurs at low prevalence, affecting approximately 1% of dogs annually within populations where rabies is endemic [6,18–20], the high seroprevalences detected in some studies (e.g. 7.4% [21]; 28.0% [22]; 28.8% [23]; 30% [24]) could suggest high rates of non-lethal exposure relative to the rate of exposure leading to clinical infection. While in some cases animals with no history of rabies exposure may test positive due to non-specific neutralisation or cross-reactivity, these high estimates of seroprevalence raise the possibility that naturally acquired immunity could play a more significant role in rabies dynamics than previously considered [16].

As a result of the low incidence of rabies in domestic dog populations, rabies seldom leads to substantial depletion of the population [19,25,26]. However, there is evidence that rabies transmission is frequency dependent, with transmission rates remaining relatively constant across a wide range of domestic dog densities [18,27]. Modelling suggests that this form of transmission should lead to high-prevalence outbreaks and substantial population losses [18]. Mechanisms that could limit rabies incidence under real-life conditions include spatial structure (which could lead to local depletion of the susceptible pool without widespread transmission), and human intervention, such as killing and isolation of infectious dogs, following increased incidence [5,9,18,28]. Naturally acquired immunity could potentially also contribute to the low-level persistence of rabies by protecting a proportion of the population, which can then produce new susceptible hosts. Naturally acquired immunity has been considered as a mechanism for the persistence of rabies in vampire bats [29], but is usually not considered in domestic dog models [e.g. 5,7,30].

In this study, we explore the implications of naturally acquired immunity to the rabies virus, resulting from subclinical exposure, for the dynamics of rabies in domestic dogs using a stochastic model parameterised for Laikipia County, Kenya. Rabies is endemic in Laikipia and a seroprevalence of 28% was previously reported in the domestic dog population [22]. A non-spatial model is initially used to explore a wide range of parameter values for naturally acquired immunity in domestic dogs. This model is then extended into a spatial model to consider a subset of potential immunity scenarios. While the spatial model relies on a greater number of assumptions, it allows for consideration of the implications of immunity under a more realistic approximation of rabies dynamics.

## Methods

A stochastic model of rabies was developed, parameterised for the domestic dog population in Laikipia County, Kenya. A compartmental structure was used with dogs divided into susceptible (S) individuals which are able to contract the virus, exposed (E) individuals which are incubating the virus, infectious (I) individuals which are able to transmit the virus, and individuals with naturally acquired immunity (R) which are immune to re-infection and are assumed to have detectable rabies antibodies. A total population size of 63,434 dogs was simulated and it was assumed that all individuals in the population were unvaccinated. Methods for estimating domestic dog numbers and simulating dog demography are presented in the S1 Text.

### Transmission dynamics

For rabies, the majority of transmission is through bite exposure [31]. However, other routes of transmission, such as through oral exposure, may be relevant when considering subclinical infection [13,32,33]. Exposure was therefore defined as any interaction between individuals that could result in viral transfer. The exposure rate was assumed to be frequency dependent, based on several studies which have indicated that R_0_ (the basic reproduction number: the average number of secondary cases produced by one case in a completely susceptible population) for rabies is relatively consistent for domestic dog populations across a range of population densities [27,34,35].

Initial model exploration was conducted using an R_0_ value of 1.2, which is in the range of one to two typically reported for domestic dog rabies [9,35]. The influence of higher R_0_ values within this range was also considered using the spatial model. The following equation for R_0_ was rearranged to calculate the infectious exposure rate (β), the number of individuals exposed per day by an infectious individual:

$$R_{0}=\beta \phi /\nu$$ (1)

The product of the exposure rate (β) and probability of developing clinical infection following exposure (ϕ) captures the rate of transmission. The basic reproduction number, R_0,_ is found by multiplying this quantity by the average duration of the infectious period (1/ν).

### Modelling naturally acquired immunity

Following exposure of a susceptible individual, we assumed there were three possibilities. Depending on the probability of developing clinical infection (ϕ), a proportion of individuals enter the exposed compartment, from which they progress to becoming clinically infectious at rate σ per day. Once infectious, the dogs succumb to rabies after 3.1 days (ν^-1^) on average [9]. The remaining proportion (1-ϕ) are subclinically exposed and either became immune through developing an antibody response (ρ), and enter the R compartment, or remain susceptible (1-ρ). This model structure makes the assumptions that dogs cannot develop symptomatic rabies and then recover and that individuals which are subclinically exposed do not transmit the disease.

In this model, it was assumed that any individuals in the immune (R) compartment had detectable rabies antibodies which confer protective immunity. Therefore, the proportion of individuals in the R compartment was considered to be equal to the predicted seroprevalence. We assumed the dynamics of antibodies developed through natural exposure would be comparable to those from vaccine-derived immunity. Based on the rate at which antibody titres become undetectable under field conditions following vaccination, for initial model exploration it was assumed individuals would remain in the R compartment for one year on average (δ^-1^) [36–38]. However, challenge experiments have shown that rabies immunity can persist for longer durations, and dogs may remain protected even where antibody tires have waned below detectable levels [39–41], therefore the influence of a longer persistence time of three years was also explored using the spatial model.

The following equations describe the full non-spatial model:

$$\frac{dS}{dt}=a(S+R)-\frac{\beta SI}{N}((1-\phi )\lambda +\phi )+\delta R-\mu S$$ (2)

$$\frac{dE}{dt}=\frac{\beta SI}{N}\phi -\sigma E$$ (3)

$$\frac{dI}{dt}=\sigma E-\nu I$$ (4)

$$\frac{dR}{dt}=\frac{\beta SI}{N}(1-\phi )\lambda -\delta R-\mu R$$ (5)

$$a=\left\{ \begin{array}{c} a\_max\frac{1-qN}{K},if\;N<K/q\\ 0,otherwise \end{array}\right.$$ (6)

Parameters values are described in Table 1.

**Table 1:** Non-spatial model parameter values. Sensitivity analysis was conducted for all parameters in the non-spatial model, excluding R_0_ and K which were kept constant, and q and β, which were calculated from other parameters. Sensitivity analysis results are presented in the S2 Text.

Epidemiological description	Symbol	Value	Notes	Source
**Basic reproduction number**	R_0_	1.2	Higher values of 1.5 and 2 were also explored using the spatial model.	[9,35]
**Maximum birth rate**	a_max	0.00302 per dog per day	Data from Laikipia County domestic dog population for sex ratio, litter size and litters per year.	[42]
**Background death rate**	μ	0.00123 per dog per day	Assumes average lifespan of 2.2 years	[9]
**Scaling parameter for relationship between birth rate and population size**	q	0.593	Parameterised so birth rate equal to death rate at carrying capacity.	
**Exposure rate per infectious individual per day**	β	-	Calculated from R_0_, ϕ and μ.	
**Probability of developing clinical infection after exposure**	ϕ	-	Varied for model exploration between 0.05 and 0.95.	
**Probability of developing protective immunity following subclinical exposure**	ρ	-	Varied for model exploration between 0 and 1.	
**Rate of progression to clinical infection**	σ	0.0448 per exposed dog per day	Reciprocal of average latent period (22.3 days).	[9]
**Rate of waning immunity**	δ	0.00274 per immune dog per day	Assumed average duration of naturally acquired immunity of 1 year. A longer duration of 3 years is also explored using the spatial model.	
**Rate of rabies mortality**	ν	0.323 per infectious dog per day	Reciprocal of average infectious period (3.1 days).	[9]
**Total carrying capacity and starting population**	K	63,434 dogs	Estimated from human-to-dog ratio.	

### Incorporating spatial structure and human intervention

While the non-spatial model allows exploration of rabies dynamics while minimising the number of assumptions made, it fails to capture key aspects of rabies dynamics. Previous studies developing rabies epidemiological models have shown the importance for rabies dynamics of spatial structure, human-mediated movement of dogs and human intervention in the case of high rabies incidence [18,43–45]. To consider the implications of naturally acquired immunity under a more realistic scenario, the non-spatial model was extended to incorporate spatial heterogeneity and human intervention.

**Fig 1:**
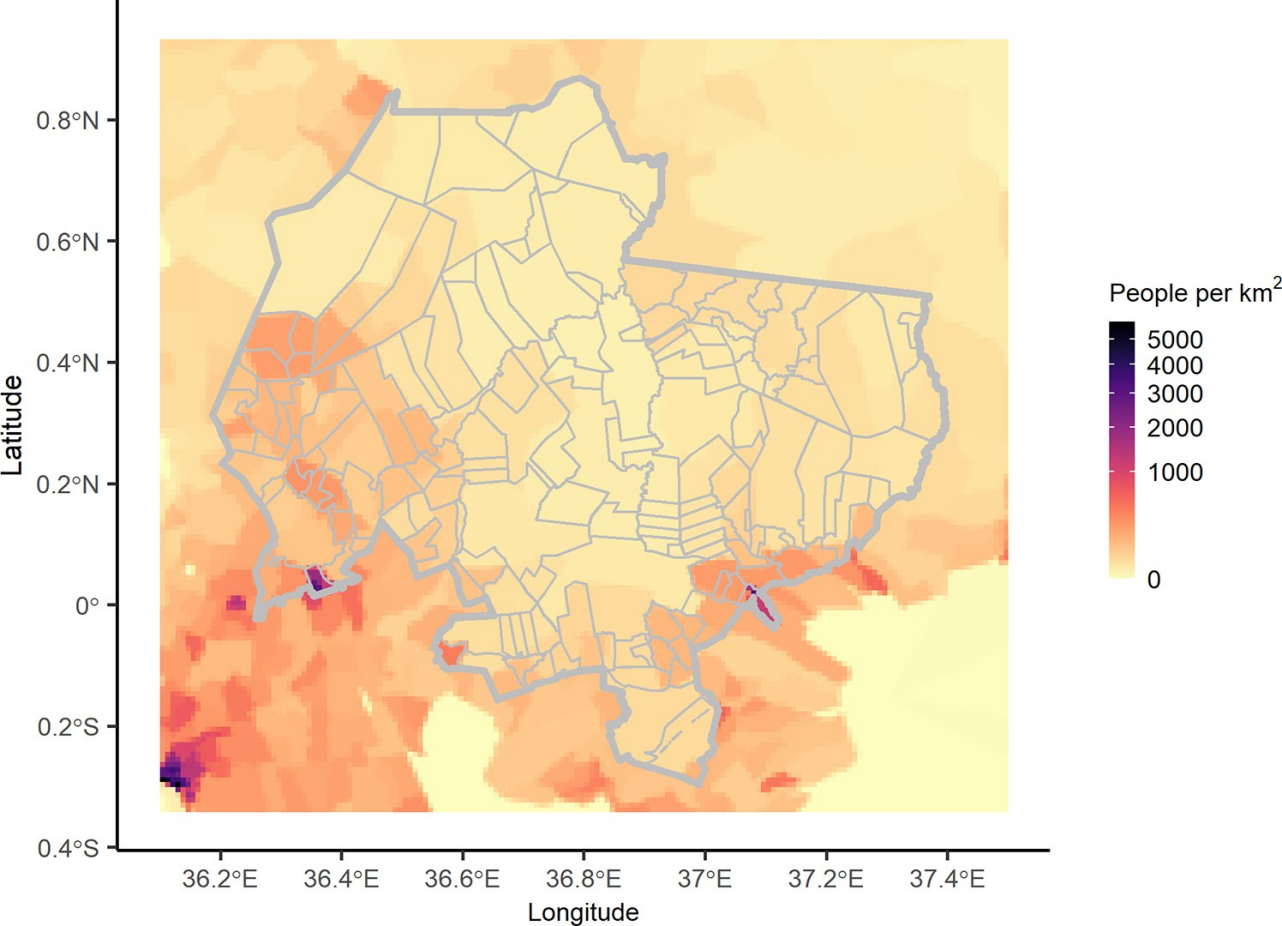
Map of Laikipia County showing underlying human density and patches used in the spatial model. Human population per square kilometre for Laikipia county and the surrounding areas is shown, based on population counts available at https://sedac.ciesin.columbia.edu/data/set/gpw-v4-population-count-rev11 [46]. Grey lines indicate patch boundaries with the thicker line indicating the border of Laikipia County. Patch boundaries were modified from a shapefile of property boundaries from Mpala Research Centre, Laikipia County, Kenya [47].

A patch structure was used, a form of metapopulation model in which sub-populations represent adjacent parcels of land. In total 154 patches were included, representing a range of land uses, such as individual ranches, villages and communities which have distinct human communities and are therefore expected to have discrete domestic dog populations (Fig 1).

Infectious dogs were assumed to remain within their patch but could transmit to susceptible individuals in other patches. The force of infection within patch *i* was therefore assumed to depend on the number of infectious individuals, multiplied by the probability of contact with the patches those infectious individuals were in. Details for calculating contact probabilities are provided in the S1 Text. While infectious dogs were assumed to remain in their patch, movement of susceptible (S), exposed (E) and immune (R) dogs between patches by human-mediated movement was simulated using a gravity model (See S1 Text for details). Human intervention in response to high rabies incidence through interventions such as tying up or killing rabid dogs has been suggested to be an important factor in limiting rabies incidence [9,18]. This response was incorporated into the model through an incidence-dependent increase in the mortality rate of infectious dogs, assumed to act at a local level within patches. Within each patch, it was assumed that if more than 1% of the carrying capacity of the patch died from rabies in the previous month, increased human intervention caused an increase in the death rate of infectious individuals. Further detail on parameterisation of this response is provided in the S1 Text, and results are presented from simulations across a range of levels of intervention to show the influence on model outputs in S1 Fig.

### Immunity scenarios

The parameters of key interest for the model are the proportion of exposures leading to clinical infection (ϕ) and, of subclinical exposures (1-ϕ), the proportion which develop immunity (ρ). Of bite exposures, Hampson et al. (2009) estimated that 0.49 led to clinical infection. However, other routes of exposure, such as feeding on infected carcasses, or saliva transfer during social contact, may be less likely to lead to clinical infection relative to bite exposure [31,33]. Compared to considering only bite exposures, incorporating other forms of exposure could therefore lead to a higher exposure rate (β) but lower probability of developing clinical infection (ϕ).

Using the non-spatial model, to explore the implications of different probabilities of non-lethal rabies exposure, we varied ϕ at five levels between 0.05 (very few exposures lead to clinical infection) and 0.95 (almost all exposures lead to clinical infection) but fixed R_0_ at 1.2. As a result, when ϕ was higher, the exposure rate (β) was lowered according to the relationship R_0_ = β ϕ/ν, to produce the same number of secondary clinical infections. This relationship gave a range for β from 0.37–7.06 exposures per infectious individual per day for an R_0_ of 1.2. For each level of ϕ, we ran the model across five levels of ρ, the probability of developing immunity, from 0 (no subclinical exposures lead to immunity) to 1 (all subclinical exposures lead to immunity) to give 25 parameter combinations in total. In addition, a sensitivity analysis of the non-spatial model is presented in the S2 Text.

Following exploration of the full range of parameter values, three parameter combinations were considered in detail, representing three potential scenarios (Table 1). The first scenario (A) assumed no development of naturally acquired immunity. In this scenario, it was assumed transmission only occurred through bite exposures and 50% of bites led to clinical infection (Based on estimate of 0.49 from Hampson et al. (2009), ϕ = 0.5) with those individuals not developing clinical infection remaining susceptible (ρ = 0). The second scenario (B) assumed immunity could be acquired following non-lethal bite exposure. The probability of clinical infection was equal to the first scenario (ϕ = 0.5) but of bite exposures which did not lead to clinical infection, it was assumed that 25% developed immunity (ρ = 0.25). The third scenario (C) represents the least conservative scenario for naturally acquired immunity. It was assumed that, in addition to bite exposure, other forms of exposure also occurred, such as oral exposure through social contact, which increased the exposure rate, but a lower proportion of exposures developed clinical infection (ϕ = 0.05 and β = 7.7). Of subclinical exposures, we assumed 50% developed immunity (ρ = 0.5).

### Running model and outputs extracted

The model was implemented using the SimInf package in R [48]. Infection dynamics were implemented as continuous-time Markov chains using the Gillespie stochastic algorithm. The model was run with a daily time step with outputs extracted at weekly intervals. For each simulation of the spatial model, the model was initiated with a prevalence of 0.5% (24 dogs) in the patch with the largest population size. In the non-spatial model, 24 individuals were also introduced. For each parameter combination, 1000 simulations were run for 30 years.

Probability of rabies persistence was measured as the percentage of simulations in which infectious individuals remained present in the population at 30 years post-introduction. For simulations in which rabies remained present, the annual incidence per 100,000 dogs for the final year, and the immune proportion in the final time step across the population were extracted. In addition, to consider spatial variation in predicted seroprevalence between patches in the spatial model, serology sampling within-patches was simulated. For each simulation, a patch was randomly selected and sampling of 30 individuals simulated. The proportion of these individuals in the R compartment was taken as the within-patch predicted seroprevalence.

### Empirical estimates for rabies incidence, population decline and seroprevalence

To consider the plausibility of different parameter combinations for non-lethal exposure and naturally acquired immunity, we compared model outputs for rabies incidence, population decline, and predicted seroprevalence to empirical estimates extracted from the literature. For Laikipia County, there is currently no estimate of annual rabies incidence in the domestic dog population. Therefore, in the absence of location-specific data, we extracted plausible ranges from other free-ranging domestic dog populations (see Table 2). We extracted these data from studies conducting active surveillance, as passive surveillance is likely to significantly underestimate incidence [26]. Based on these studies, we assumed an upper limit for annual rabies incidence of 1,500/100,000 (Table 2).

**Table 2:** Parameter values for naturally acquired immunity scenarios A, B and C.

Scenario	Exposure rate per infectious individual (β)	Probability of developing clinical infection (ϕ)	Probability of developing immunity (ρ)	Probability that exposure leads to immunity ((1-ϕ)ρ)
A- No immunity	0.79 per day	0.5	0	0
B- Low immunity	0.79 per day	0.5	0.25	0.1275
C- High immunity	7.74 per day	0.05	0.5	0.1875

For each of these three immunity scenarios, the influence of a longer persistence time for naturally acquired immunity (δ^-1^ = 3 years) and higher R_0_ values of 1.5 and 2 were also explored.

Due to the low incidence of rabies, substantial population decline is not expected to result from endemic rabies, although populations may fluctuate in size. For example, in dog populations with endemic rabies in Indonesia and South Africa, Morters et al. (2014) report variation in population size of up to 22% from the mean over the study period. In South Africa, Conan et al. (2015) report annual changes in population size from +18.6% to -24.5%. In this study we assumed that once endemic, taken as 30-years post-introduction, rabies would not cause population decline greater than 20% relative to the carrying capacity.

We also considered the seroprevalence predicted by the model relative to empirical estimates. In Laikipia, Prager et al. (2012) reported a seroprevalence of 28% in 75 domestic dogs tested using a rapid fluorescent focus inhibition test (RFFIT; 95% CI: 18.2–39.6%). However, this study used a low cut-off of 0.05 IU/mL which increases the probability of false positives. There is also evidence that enzyme-linked immunosorbent assays (ELISAs) are more specific for detecting non-lethal exposure relative to neutralisation tests [21]. Studies using ELISAs have also detected high seroprevalences. Laurenson et al. (1997) found a seroprevalence of 30.0% in Namibia [24], Bahloul et al. (2005) of 28.8% in Tunisia [23] and Cleaveland et al. (1999) of 7.4% in Tanzania [21]. We therefore considered a range of 7–30% for seroprevalence in domestic dogs [16].

## Results

### Non-spatial model

**Fig 2:**
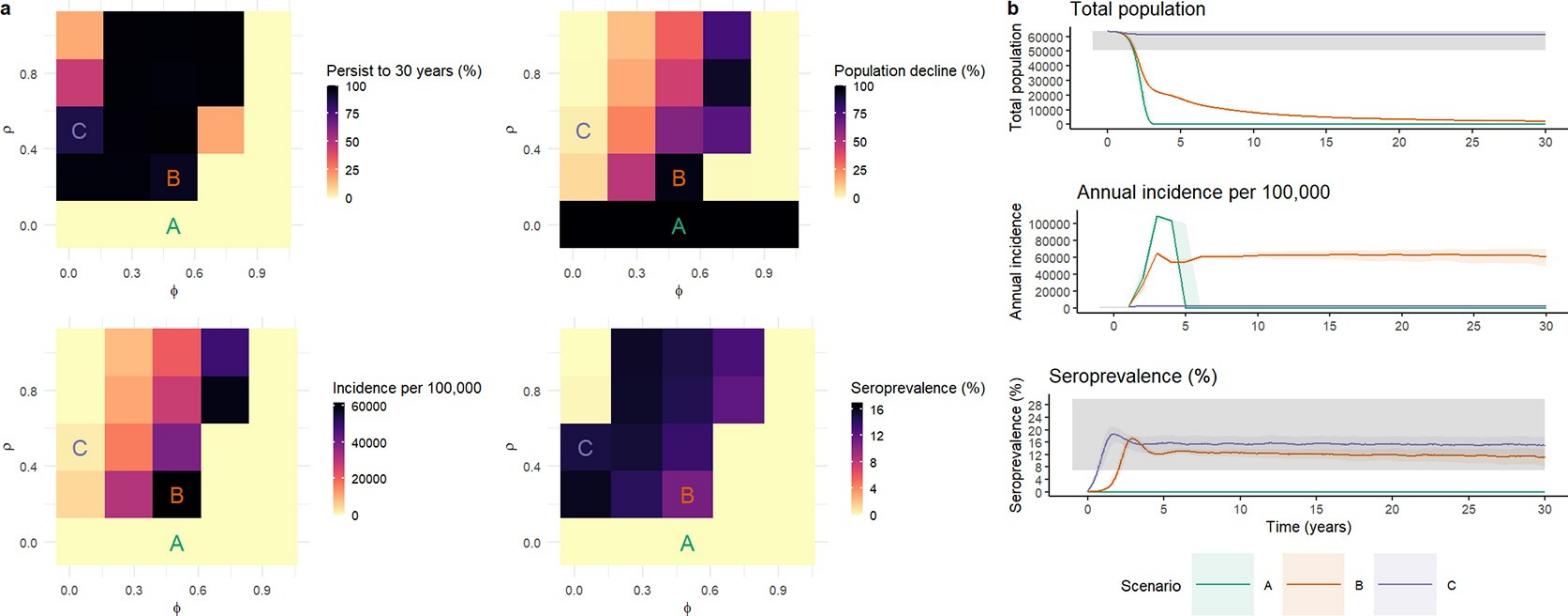
Results from non-spatial model of effects of non-lethal exposure (ϕ) and acquired immunity (ρ) on probability of rabies persistence, median percentage population decline, median annual incidence per 100,000 and median predicted percentage seroprevalence. Plot a presents the median values for the full range of parameter combinations. Plot b shows a subset of parameter combinations (Scenarios A, B and C) indicated by labels on plot a with median values and interquartile ranges over the full 30 years for total population size, annual incidence per 100,000 dogs and seroprevalence (%). Grey bands indicate the range of values considered plausible based on empirical estimates, as shown in Table 3.

Assuming an R_0_ of 1.2, with no spatial structure and no incidence-dependent human response, rabies was not predicted to persist in the absence of any naturally acquired immunity (ρ = 0) or with a low probability of sub-clinical exposure (ϕ = 0.95) (Fig 2A). Allowing a proportion of dogs to develop immunity (ρ>0) increased the probability of rabies persistence, with the highest persistence probabilities at intermediate probabilities of acquired immunity. For example, assuming 50% of exposures led to clinical infection, with 25% of sub-clinically exposed individuals developing immunity (Scenario B- ϕ = 0.5, ρ = 0.25), rabies remained endemic in 94% of the simulations. The median annual incidence under this scenario was high at 61,820/100,000 (Interquartile range (IQR): 19,139/100,000), with a high population decline relative to the carrying capacity (Median: 96.0%, IQR: 2.0%). Assuming higher probabilities of acquired immunity led to lower persistence, due to reduced incidence and an increased probability of stochastic extinction. For example, under scenario C, with a low probability of an individual developing clinical infection (ϕ = 0.05), and a high probability of developing immunity (ρ = 0.5), incidence was reduced to 2,207/100,000 (IQR: 1181) with rabies persisting in 87% of simulations. No parameter combination in the homogenous model led to median outputs for both incidence and population decline in the range considered plausible (Table 3). However, of the parameter combinations in which rabies was predicted to persist in the majority of simulations, Scenario C (the high immunity scenario, Table 2) had incidence closest to the plausible range and a low median population decline of 3.6% (IQR: 1.1%) (Fig 2).

**Table 3:** Assumed plausible ranges of population decline, annual incidence per 100,000 dogs and seroprevalence for comparison to model outputs.

Outputs	Assumed plausible range	Literature estimates and sources
Population decline relative to carrying capacity	0–20%	[49,50]
Annual incidence per 100,000 dogs	<1500/100,000 dogs, allowing for under-reporting	860/100,000 - [26]
		1089/100,000 - [20]. For unvaccinated villages where active surveillance conducted.
		412/100,000 - [19]
Seroprevalence	7–30%	[16]

Of the full set of 25 parameter combinations considered, the combination which generated the highest median predicted seroprevalence, of 16.0% (IQR: 3.5%), was a probability of an individual developing clinical infection (ϕ) of 0.05 and a probability of developing immunity (ρ) of 0.25. This predicted seroprevalence is close to the threshold for herd immunity for an R_0_ of 1.2 (1-1/R_0_ = 0.167). At this threshold each infectious individual infects on average one other, leading to stable endemic infection. For the subset of three immunity scenarios considered, predicted seroprevalences using the non-spatial model were 0.0% (IQR: 0.0%), 11.1% (IQR: 5.2%) and 14.9% (IQR: 6.1%) for scenarios A, B and C respectively.

### Incorporation of spatial structure and incidence-dependent human response

**Fig 3:**
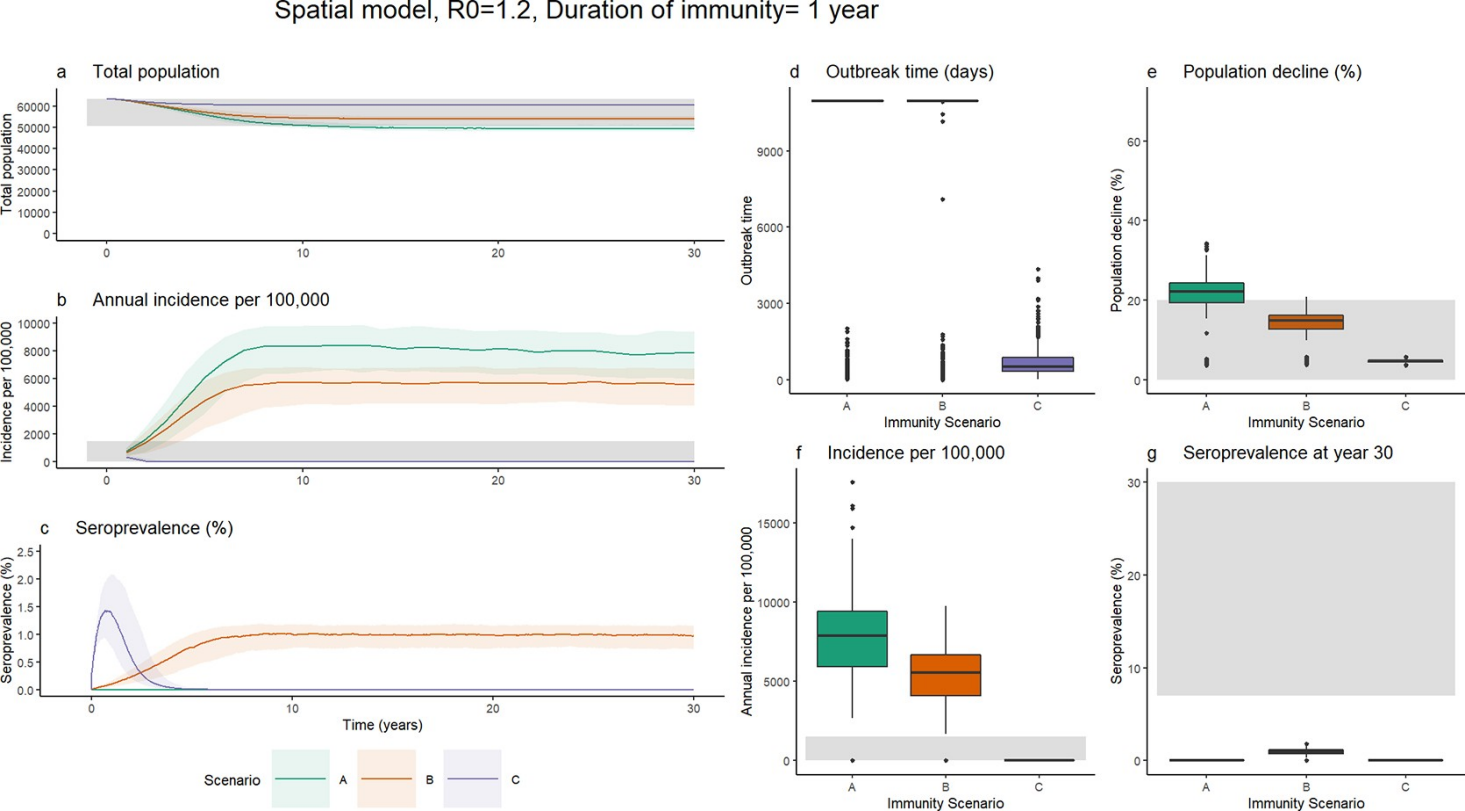
Results from the three immunity scenarios for the spatial model with R_0_ = 1.2 and a 1-year duration of naturally acquired immunity. Median and interquartile range for the total population size, annual incidence and seroprevalence for each of the immunity scenarios over the 30-year simulation are shown in a-c respectively. **Fig 3d–3g** shows boxplots for the outbreak time (d) (time from introduction to no infectious individuals remaining, or end of simulation), population decline (e) relative to the carrying capacity, incidence per 100,000 dogs in year 30 post introduction (f) and predicted seroprevalence at the end of the simulation (g). Grey bands indicate the range of values considered plausible based on empirical estimates, as shown in Table 3.

The non-spatial model was extended to a spatial patch model including human-mediated movement of dogs and incidence-dependent human response to explore the three immunity scenarios (Fig 3). Results from spatial model formulations excluding human-mediated movement and incidence-dependent human response are presented in S2 and S3 Figs.

Incorporating these additional assumptions resulted in an increase relative to the non-spatial model in the probability of persistence of rabies in the absence of immunity, with the disease persisting in 82.9% of simulations for scenario A. The persistence probability associated with scenario B, with low levels of naturally acquired immunity was marginally lower, at 81.9%. In the high immunity scenario, C, rabies did not persist in any simulation at this R_0_ value. Scenario C assumed a high transmission rate and for every exposure leading to clinical infection (ϕ = 0.05), 9.5x more become immune ((1-ϕ)ρ where ρ = 0.5). As a result, rapid depletion of the susceptible population within patches reduced the probability of persistence.

For both scenarios A and B, median incidence was higher than the range considered plausible, however incidence was lower in the low immunity scenario (B) relative to the no immunity scenario (A), with a median of 5,566/100,000 (Scenario A, IQR: 2,583/100,000) relative to 7,810/100,000 (Scenario B, IQR: 3,485/100,000; Fig 3). Median population decline for scenario B was within the plausible range, at 15% (IQR: 3%), whereas without immunity (Scenario A) decline was greater at 22% (IQR: 5%). In the low immunity scenario (B), seroprevalence at a population-wide level was predicted to be low, with a median of 0.97% (IQR:0.42%). Incorporating low levels of naturally acquired immunity therefore lowered the predicted incidence and mitigated the population decline, despite a low population-wide predicted seroprevalence.

**Fig 4:**
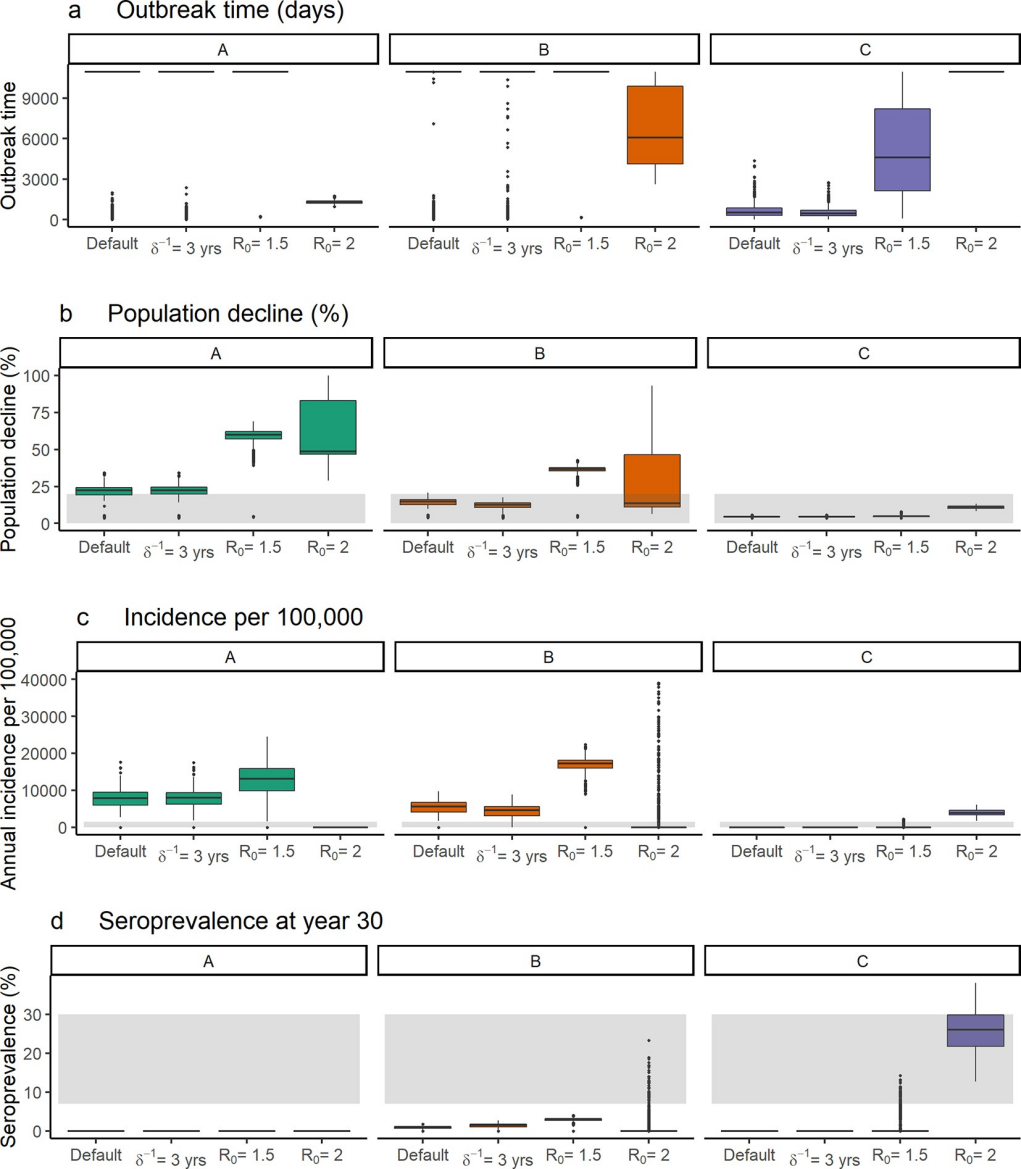
Comparison of spatial model outputs with increased average duration of naturally acquired immunity (δ^-1^ = 3 years) and higher R_0_ values (R_0_ = 1.5 and R_0_ = 2), compared to the default model with R_0_ = 1.2 and a 1-year average duration of immunity. Boxplots are shown for the outbreak time (a) (time from introduction to no infectious individuals remaining, or end of simulation), population decline relative to the carrying capacity (b), incidence per 100,000 dogs in year 30 post introduction (c) and seroprevalence at the end of the simulation (d). Grey bands indicate the range of values considered plausible based on empirical estimates, as shown in Table 3.

The duration of naturally acquired immunity for rabies has not been established, and primary analyses assumed an average duration of 1 year, based on the persistence of detectable antibodies following vaccination from field studies [36–38]. However, increasing this duration to three years (δ = 1/1095), did not substantially change the model outputs (Fig 4). For scenario B, increasing the duration of immunity increased median seroprevalence only slightly, from 0.97% (IQR:0.42%) to 1.5% (IQR:0.7%). Due to the rapid turnover in domestic dog populations, with the average lifespan assumed to be 2.2 years (μ^-1^), seropositive dogs are likely to die before immunity wanes, therefore limiting the influence of longer persistence of naturally acquired immunity.

Assuming higher R_0_ values of 1.5 and 2 led to substantial changes in the model outputs (Fig 4). Increasing R_0_ led to a higher persistence probability for the high immunity scenario, C, with rabies persisting in 13.8% of simulations for an R_0_ of 1.5 and 89.3% for an R_0_ of 2, relative to 0.0% for an R_0_ of 1.2. In simulations for scenario C where rabies did persist, the median incidence for an R_0_ of 1.5 was 1141/100,000 (IQR: 631/100,000) and population decline was 6.5% (IQR: 0.8%), consistent with the ranges considered plausible (Fig 4). For an R_0_ of 2, the incidence was higher at 3904/100,000 (IQR: 1313/100,000), however the population decline remained within the plausible range at 8.9% (IQR: 1.7%). The median predicted seroprevalence for these scenario C simulations was 6.8% (IQR: 3.8%) for an R_0_ of 1.5, and 26.1% (IQR: 8.1%) for an R_0_ of 2. For scenarios A and B, increasing R_0_ to 1.5 led to higher incidence and greater population decline, further increasing these outputs above the levels considered plausible (Fig 4). Further increasing R_0_ to 2 led to rabies not persisting under the no immunity scenario (A) in any simulations, and only persisting in 20.5% of simulations under the low immunity scenario (B).

### Spatial variation in predicted seroprevalence

**Fig 5:**
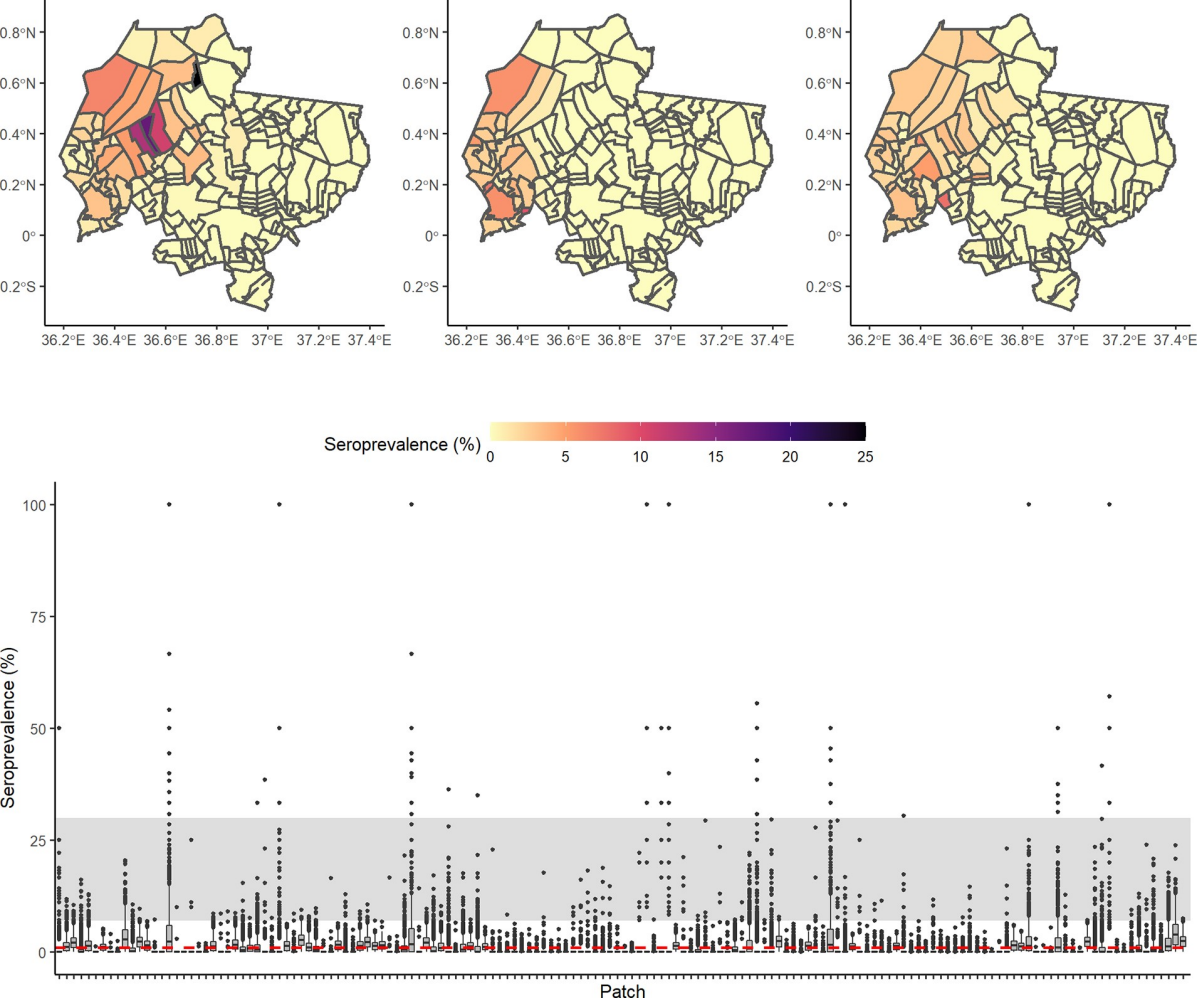
Spatial variation in seroprevalence between patches from model simulations for scenario B, with R_0_ = 1.2 and δ = 1/365. a) Maps of Laikipia County showing seroprevalence in each patch at 30 years post introduction from three randomly selected simulations. Patch boundaries were generated from a shapefile of property boundaries from Mpala Research Centre, Laikipia County, Kenya [47]. b) Boxplots showing variation in seroprevalence in all patches at 30 years post introduction. The grey band indicates the range of seroprevalence considered plausible based on empirical estimates, as shown in Table 3. The red dotted line indicates the median population-level seroprevalence.

At a population level, predicted seroprevalences for an R_0_ of 1.2 were low. However, substantial spatial variation in seroprevalence between patches occurred, as shown in Fig 5. Simulating sampling of 30 individuals from a single randomly selected patch for each of the simulations for scenario B gave a median predicted sample seroprevalence of 0. However, for the 11.1% of simulations in which seropositives were detected in the sample, the median predicted seroprevalence was 3.3%, with a maximum of 13.3%, relative to the population level seroprevalence of 0.97% (IQR:0.42%). Therefore, depending on the distribution of sampling, sample seroprevalences may vary substantially from the population-level seroprevalence.

## Discussion

Immunity in models of canine rabies is typically only considered in relation to vaccination. However, rabies-specific antibodies in unvaccinated individuals have been detected in a number of studies of domestic dog populations. While there is still debate over the importance of these antibodies [16], the high seroprevalences detected in some studies warrant consideration of their potential implications. In this study, a model of domestic dog rabies was developed to explore the influence of subclinical infection and immunity for rabies dynamics.

Exploration of the non-spatial model showed that, in the absence of other factors limiting incidence of rabies, naturally acquired immunity could play a role in stabilising rabies outbreaks. Without spatial structure and assuming rabies transmission was frequency dependent, with an R_0_ value of 1.2, introduction of rabies was predicted to lead to depletion of the domestic dog population in the absence of immunity. In previous non-spatial models of rabies, population depletion has been prevented by assumptions of density-dependent transmission, or low R_0_ values with high population growth [6,18,30]. Studies of the transmission dynamics of rabies have suggested these assumptions are potentially unrealistic, with evidence for higher R_0_ values and frequency-dependent transmission [9,18,27,34,35]. Our model suggests that naturally acquired immunity can facilitate outbreak persistence under these assumptions by reducing incidence and preventing depletion of the susceptible population, as immune hosts produce susceptible offspring which can then become infected. However, having high rates of immunizing subclinical exposure could also push incidence to low levels, leading to an increased probability of stochastic fade out of the outbreak. The non-spatial model therefore predicted that endemic infection was most likely at intermediate levels of acquired immunity.

While the non-spatial model allowed exploration of the implications of acquired immunity with limited assumptions, it fails to capture key aspects of rabies dynamics. Previous studies have indicated the importance of spatial structure, human-mediated dog movement and human response to increased rabies incidence as important factors in rabies dynamics [4,18,28,51,52]. Results from the spatial model incorporating these factors showed that persistence of rabies could occur in the absence of immunity, as spatial structure and human intervention act to limit incidence and prevent population extinction. However, model predictions for annual incidence and population decline remained higher in the absence of immunity than empirical estimates. Including finer-scaled spatial structure and higher levels of human intervention could produce realistic estimates in the absence of immunity. For example, Beyer (2010) considered spatial structure on the scale of a single village of 288 dogs [28], considerably lower relative to the total carrying capacity of 63,434 dogs in this model. This fine-scale structure led to realistic outputs without inclusion of immunity. Furthermore, assuming a stronger response to high rabies incidence through human intervention also led to lower incidence in the absence of immunity (S2 Fig). Estimating the strength of this response is highly challenging. Hampson et al. (2009) found that in Tanzania, killing of infectious dogs reduced the infectious period by around 16%. However, how this response scales with incidence and varies temporally and spatially is unclear. Including this relationship prevents unrealistically high incidences, however as the strength of this effect has not been reliably estimated, its influence relative to other factors, such as spatial structure and immunity, is difficult to establish. While including finer scaled spatial structure or stronger human intervention could reduce incidence to levels within the range considered plausible, these factors do not account for the seroprevalences reported in empirical studies [16]. Including immunity for an R_0_ of 1.2 led to lower incidences, despite low predicted seroprevalence relative to observed levels. Potentially, naturally acquired immunity, in combination with spatial structure and human intervention, may therefore act to dampen rabies outbreaks, leading to low-level endemic infection.

For an R_0_ value of 1.2, none of the scenarios considered produced the levels of incidence, population decline and seroprevalence considered realistic. In the high immunity scenarios, rabies persistence was not predicted to occur due to depletion of the susceptible population. For an R_0_ of 1.2, herd immunity within a patch is effectively reached once more than 16.7% (1-1/R_0_ = 0.167). of the population is immune. Local susceptible depletion due to immunizing exposure can therefore lead to substantial reductions in transmission, despite low population-level seroprevalences. However, assuming higher R_0_ values of 1.5 and 2 led to persistence of rabies in 13.8% and 89.3% of simulations respectively under the high immunity scenario, as the threshold to reach herd immunity is higher. For an R_0_ of 1.5, the estimates of incidence and population decline under this scenario were also consistent with empirical estimates. Assuming higher transmission rates also led to higher median predicted population-level seroprevalences at 6.8% and 24.7% for R_0_ values of 1.5 and 2 respectively. These estimates are closer to levels observed in empirical studies, for example Cleaveland et al. (1999) reported a seroprevalence of 7.4% in Tanzania and Bahloul et al. (2005) of 28.8% in Tunisia. The higher R_0_ values remain within the range of one to two typically reported for canine rabies [9]. However, under the high immunity scenario, much higher rates of exposure, and lower probabilities of developing clinical infection, were assumed than are typically considered for canine rabies. For example, for an R_0_ of 2 under the high immunity scenario, a rate of 12.9 exposures per infectious dog per day was assumed, with only 5% of these exposures leading to clinical infection. This parameterisation for the high immunity scenario is comparable to what has been assumed for lyssavirus dynamics in bats, in which the probability of developing immunity is considered to be much higher [29,53]. However, Hampson et al. (2009) estimated that on average, a rabid dog bites 2.15 others during its infectious period, of which 49% develop clinical infection, suggesting an exposure rate of 12.9 per day is unrealistically high if bites are considered the sole source of exposure. If the high empirical seroprevalences detected in some populations are due to higher rates of exposure, it may suggest that routes of transmission other than bite exposure are leading to development of an antibody response, such as through oral exposure during social contact or feeding on infected carcasses [12,13,54].

While high seroprevalences detected in empirical studies could reflect higher transmission rates, other factors may also be responsible. Substantial variation between serology tests has been shown, with evidence that neutralisation tests such as the rapid fluorescent focus inhibition test (RFFIT) may be less specific for detecting non-lethal exposures relative to ELISAs [21]. For example, the high seroprevalence of 28% detected in Laikipia, using the RFFIT and a low cut-off, may be partially explained by false positives [22]. However, even studies using ELISAs with higher cut-offs have found high seroprevalences [23,24]. For example, Cleaveland et al. (1999) found a seroprevalence of 7.4% in a domestic dog population in Tanzania using an ELISA and no false positives were detected on a rabies-free island using this test, suggesting high specificity. A further possibility is that cross-reactivity is occurring with other circulating lyssaviruses, although in the absence of further serological and surveillance data, this cannot be confirmed [16]. A further factor which may contribute to the discrepancy between observed seroprevalences and predicted population-level seroprevalence is spatial variation. As shown in Fig 5, predicted seroprevalences were not consistent across the landscape, therefore the population-wide predicted seroprevalence is expected to differ from sample seroprevalences. For example, localised sampling in areas where there has been a recent outbreak could lead to higher detected empirical seroprevalences. However, under the low-immunity scenario, the highest simulated sample seroprevalence was 13.3%, remaining lower than reported in some empirical studies [16]. Furthermore, empirical studies have in most cases used larger sample sizes, and in a range of locations, which should lead to a closer approximation of the population-level seroprevalence [e.g. 21,22].

A further challenge to the interpretation of rabies serology is that it is currently unknown whether antibodies from natural exposure confer protection to re-exposure [16]. There is limited evidence that previously unvaccinated seropositive individuals show an anamnestic response to vaccination consistent with immunity [15,23,38,55]. However, even in vaccinated individuals, serology status is not definitive proof of an effective immune response, and individuals with a detectable titre may still succumb to the disease [56]. If antibodies from non-lethal exposure provide no, or only partial protection, this would influence the expected dynamics. For example, partial immunity could potentially allow higher seroprevalences to occur within populations without the same reduction in transmission, potentially increasing the probability of persistence at higher seroprevalence levels.

The results of this study should be considered in the context of rabies surveillance and control. In this study we assumed all dogs were unvaccinated, to allow consideration of the influence of naturally acquired immunity on persistence in the absence of control. In Laikipia, mass vaccination has only been conducted since 2015 and prior to this point by far the majority of dogs would have been unvaccinated [57]. However, low levels of vaccination coverage may act in a similar way to naturally acquired immunity, dampening incidence of rabies and potentially promoting persistence. Kitala et al. (2002) modelled coverage of 24% in Machakos district, Kenya and found that this low level of coverage increased the stability of transmission and led to endemic establishment. While not within the scope of this paper, future work to include both vaccination and non-lethal exposure could lead to further insights into the influence of naturally acquired immunity in domestic dog populations in the context of control strategies. Vaccination may reduce the predicted influence of naturally acquired immunity on dynamics, as the resulting reduction in transmission will reduce exposure and therefore the expected seroprevalence. As a result of this, and the uncertainty over the implications of unvaccinated seropositives, high seroprevalence within a population should not be taken as evidence that lower vaccination coverage is required to reach herd immunity and eradicate rabies. Findings from this study also bring into question the use of serology for rabies surveillance. While serology could provide a mechanism to conduct rabies surveillance that does not rely on reporting of clinical cases, the challenges of interpreting rabies serology, in addition to the cost of implementation, limits the feasibility of this strategy. However, given the number of studies reporting high rabies seroprevalence, across a wide geographical range [16], the potential for high rates of transmission leading to sub-clinical exposure should be considered, in particular as higher R_0_ values may indicate the need for higher vaccination coverage for effective control.

There were a number of limitations to the modelling methods used. In the model, Laikipia was treated as an isolated unit. In reality, the county is continuous with other areas with domestic dog populations. Potentially, persistence of rabies occurs at a wider scale with introductions of rabies from other populations sustaining the disease within Laikipia, as shown in domestic dog populations in N’Djamena, Chad and Bangui, Central African Republic [43,45]. Further data are required to improve parameterisation of human-mediated dog movement, to assess the influence of both importation from outside the county and movement of dogs within the county [4]. In the model, it was also assumed that contact between infectious individuals in different patches was determined by distance. In reality, physical features and human geography will be influential for contact, for example depending on the presence of roads linking properties, or physical barriers such as fencing or rivers separating them [27]. A further limitation was the failure to capture heterogeneity in certain parameters. Rabies transmission is highly heterogeneous with most individuals not transmitting at all, whereas others infect large numbers of other individuals [9]. A negative binomial distribution has been suggested to be the best fit to model these dynamics [18], however the use of the Gillespie algorithm to model stochasticity precluded fitting specific distributions. Variation in the duration of the latent period has also been shown to influence rabies persistence [4]. Including this heterogeneity may have increased the probability of persistence of rabies.

In conclusion, our results suggest that subclinical immunizing exposure could play a role in the dynamics of rabies in domestic dogs, limiting disease incidence and population decline. However, consideration of other factors, such as spatial structure and human response to rabid dogs, is also required to approximate realistic rabies dynamics. The scenarios for naturally acquired immunity explored for an R_0_ of 1.2 produced low predicted seroprevalences relative to those observed in some empirical studies. Higher seroprevalences could be explained by higher rates of immunizing subclinical infection within domestic dog populations, however false positives or spatial variation in seroprevalence may also contribute. If high seroprevalences do indicate high transmission rates, this supports the need for high vaccination coverage to effectively control this disease.

## Supporting information

S1 Text Additional details on model parameterisation. (PDF)

S2 Text Methods and results for sensitivity analysis of the non-spatial model. (PDF)

S1 Table Model results for non-spatial model across all 25 parameter combinations, presented as heat maps in Fig 2. (PDF)

S1 Fig Influence of incidence-dependent human response on model outputs in the absence of naturally acquired immunity (Scenario A). (PDF)

S2 Fig Comparison of model outputs from immunity scenarios (A, B and C) across different model formulations for R0 = 1.2. (PDF)

S3 Fig Comparison of model outputs from immunity scenarios (A, B and C) across different model formulations for R0 = 1.5. (PDF)

S1 Code This file contains: R script to generate the events data frame for human-mediated dog movement, an R script to run model simulations for both the non-spatial and spatial model, a csv. file containing the contact matrix for the spatial model and the shapefile of patches used. (ZIP)
