# Supplementary material for: Modelling the influence of naturally acquired immunity from subclinical infection on outbreak dynamics and persistence of rabies in domestic dogs
Source: PLoS Negl Trop Dis. 2021 Jul 20;15(7):e0009581. doi: 10.1371/journal.pntd.0009581 (PMC8330898; doi:10.1371/journal.pntd.0009581)
Supplement: S1 Text — (PDF) [file pntd.0009581.s001.pdf]

S1 Text

Estimating number of dogs

The number of dogs was extrapolated from the human population density for Laikipia County, Kenya. The total human population in Laikipia County was estimated to be 518,560 in 2019 [1]. To allow for development of a spatial model, and to account for differing human-to-dog ratios in rural and urban areas, the county was divided into individual parcels of land using a shapefile of land use types from Mpala Research Centre. Parcels with an area less than $10km^2$ were merged with adjacent neighbours. This gave a final shapefile of 154 individual parcels, ranging in size from 10.1 km^2 to 386 km^2. A raster of human density was then used to estimate the human population size in each land parcel [2]. The numbers of dogs in each parcel was estimated using a human-to-dog ratio of 7.4:1 for rural areas, defined as those with human density less than or equal to 1000 per km^2, and 21.2:1 for urban areas, those with human density greater than 1000 per km^2 [3]. Using this method, the total domestic dog population for the county was estimated to be 63,434 with an average of 412 individuals per patch (Range 5-2442). We assumed the population was currently at carrying capacity, and therefore the estimated population was used as the starting number of susceptible individuals and the total carrying capacity.

Modelling dog demography

Domestic dog populations differ from wildlife in that population growth is primarily managed by human demand, rather than resource availability [4]. In Laikipia, control of the domestic dog population is primarily through preferentially keeping male dogs and giving away or killing unwanted puppies [5]. To account for the influence of human intervention we assumed that population size was regulated primarily by the rate of introduction of new susceptible individuals into the population (a) represented by the following equation:

$$a = \begin{cases} a_{\max}\frac{1-qN_i}{K_i}, if\ N_i < K_i/q \\ 0, otherwise \end{cases} \quad (1)$$

This introduction rate was dependent on the birth rate (a_max) and human intervention, which was assumed to increase as the population size (N_i) increased relative to the carrying capacity (K_i) in each patch. This relationship was parameterised so that the introduction rate (a) declined to be equal to the background death rate (b) at the carrying capacity but is prevented from going below zero. The maximum introduction rate was assumed to be equal to the birth rate (a_max) which was estimated from data for the Laikipia domestic dog population from Woodroffe et al. (2011) [5]. This value was assumed to be equal to the estimated proportion of the population that is female (0.288), multiplied by the number of litters per female per year (0.78) and the mean litter size (5.06). This low proportion of females in the population reflects that male dogs are preferentially kept. Due to the short period for which individuals are assumed to be exposed or infectious (22.3 days and 3.1 days respectively [6]), individuals in the E and I compartments were assumed not to reproduce and not to die from causes other than rabies.

Spatial model

The following ordinary differential equations describe the dynamics of the spatial model in patch i:

$$\frac{dS_i}{dt} = a(S_i + R_i) - \frac{\beta S_i}{N_i} \Sigma p_{ij} I_j \big((1-\phi)\rho + \phi\big) + \delta R_i - \mu S_i \quad (2)$$

$$\frac{dE_i}{dt} = \frac{\beta S_i}{N_i} \Sigma p_{ij} I_j \phi - \sigma E_i \quad (3)$$

$$\frac{dI_i}{dt} = \sigma E_i - \nu w I_i \quad (4)$$

$$\frac{dR_i}{dt} = \frac{\beta S_i}{N_i} \Sigma p_{ij} I_j (1-\phi)\rho - \delta R_i - \mu R_i \quad (5)$$

$$\frac{dM_i}{dt} = \nu w I_i - r M_i \quad (6)$$

where $a = \begin{cases} a\_max \frac{1-qN_i}{K_i}, if\ N_i < K_i/q \\ 0, otherwise \end{cases}$ (7)

and $w = \begin{cases} 1,\ if\ M_i < K_i/100 \\ 1.2\ , otherwise \end{cases}$ (8)

Parameters specific to the spatial model are described in Table A.

Table A- Spatial model specific parameter values

Epidemiological description	Symbol	Value	Notes
**Probability of between patch contact**	$p_{i,j}$	Mean=0.006	
**Patch-specific carrying capacity**	K_i	Mean= 412<br>Range= 5-2442	Estimated from human-to-dog ratio.
**Incidence-dependent human response**	w	1.2	Parameterised so that when more than 1% of the carrying capacity of a patch are killed by rabies within a month, the mortality rate increases so that the effective reproductive number, the average number of secondary cases per infectious case (R_e), is less than 1 for an R_0 of 1.2. Influence of varying this parameter is shown in S1 Fig.
**Number of dogs killed by rabies in month prior**	M	-	Compartment to keep track of within-patch incidence in prior month.
**Rate at which dogs are removed from M compartment**	r	0.033	Dogs removed after 30 days on average.

Generating probability of contact between patches

To produce the probability matrix for contact between patches, within each patch 100,000 random points were generated to represent potential locations of infectious dogs. This method made the assumption that dogs are randomly distributed within patches. For each of these primary cases a location was then generated for a secondary case. Secondary cases were defined based on their distance and direction relative to primary cases. The angle of movement was generated from a uniform distribution. The distance from primary to secondary cases was sampled from a gamma distribution for transmission distance from Hampson et al. (2009) with shape and rate parameters of 0.215 and 0.245 respectively (Mean= 0.88 km, Variance= 3.58 km^2). For each secondary case the patch it fell into was identified. The proportion of secondary cases was then used as the measure of contact between different patches. Fig A shows an example of 300 points generated from these distributions to illustrate the method used.

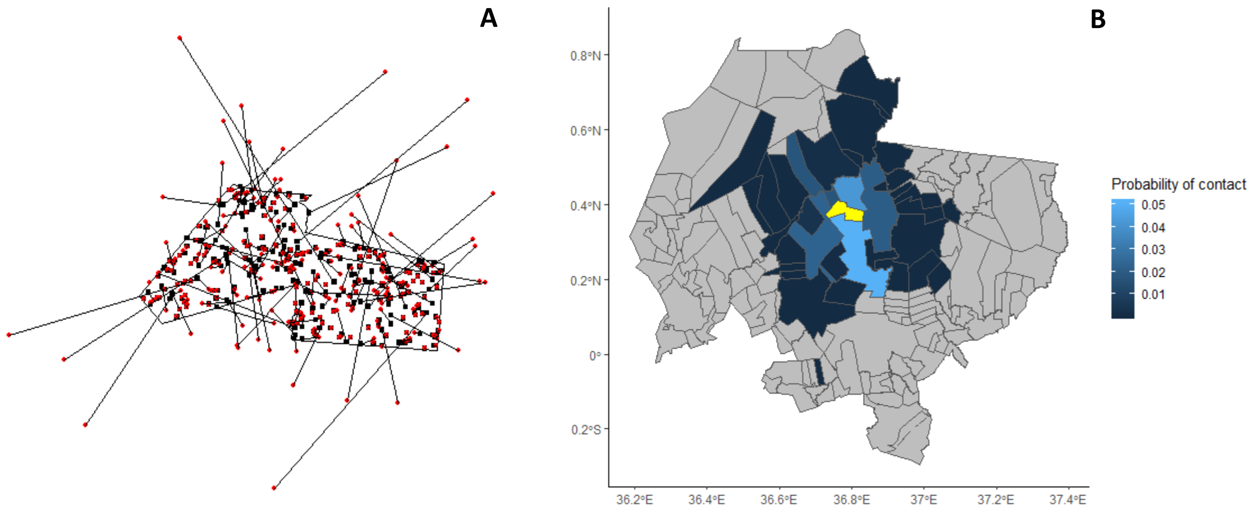

Fig A- Illustration of method of developing contact matrix for patches in Laikipia. *A) Example of 300 points generated within patch. Black squares indicate initial locations, and red circles the secondary cases. B) Map of Laikipia County showing example of contact probabilities between patches. The yellow polygon shows the patch shown in panel A and the colour of the surrounding polygons shows the probability of contact. Grey indicates the probability of contact is 0. Patch boundaries were generated from a shapefile of property boundaries from Mpala Research Centre, Laikipia, Kenya [8].*

Human mediated transport of domestic dogs

Movement of rabid dogs is limited to local-scale movements within a few kilometres [6,9]. Spatial dispersal of rabies over a larger scale is therefore thought to be influenced primarily by human-mediated movement. While symptomatic dogs are unlikely to be moved, movement of individuals in the latent phase can introduce infection to new areas. Human-mediated movement between properties of susceptible (S), exposed (E) and naturally immune (R) individuals was therefore incorporated into the model to account for this spatial spread. Movement was simulated as an event in the model occurring at weekly intervals. Data on human-mediated movement of domestic dogs are scarce. In the Central African Republic, Colombi et al. (2020) estimated a probability of 0.0046 of medium range movement (20-100km) per day for domestic dogs [9]. Ferguson et al. (2015) estimated that in the Philippines, approximately 0.2% of dogs are moved within municipalities daily. In the absence of data specific for

Laikipia, we assumed that on average 0.5% of dogs in the S, E and R compartments in each patch would move weekly. A gravity model [10,11] was used to determine the probability of movement to each other patch with the probability of movement between patch i and j ($T_{i,j}$) in a week proportional to:

$$T_{i,j} = k\frac{HP_i \cdot HP_j}{{D_{ij}}^2} \quad (9)$$

where HP_i is the human population size of patch i and $D_{i,j}$ is the distance between the centroids of patches i and j. The constant, k, was parameterised so that the mean probability of a dog leaving a patch in a given week was 0.5%. S2 Fig and S3 Fig show model results without human-mediated domestic dog movement included, for R0 values of 1.2 and 1.5 respectively.

Incidence-dependent human response parameterisation

Human response to high rabies incidence, for example through tying up or killing symptomatic individuals, has been suggested to play a role in limiting rabies incidence [12,13]. Incorporating this response into models can prevent unrealistically high incidences occurring. However, parameterising this response is challenging as in reality it would be dependent on a number of factors, for example the probability of detecting rabid dogs. In the model, we parameterised this response as an increase in the mortality rate for infectious individuals within patches once incidence within the patch crossed a threshold. To keep track of the recent incidence within patches, an additional compartment (M) was included in the model into which infectious individuals entered after succumbing to infection and left after 30 days on average. When the number of individuals in this compartment was greater than 1% of the carrying capacity ($M_i/K_i>100$), it was assumed there would be an increase in the mortality rate for infectious individuals of w. This response was parameterised so that the effective reproduction number was decreased to below one for an R_0 of 1.2.

References

1. Kenya National Bureau of Statistics. 2019 Kenya Population and Housing Census Volume I: Population by County and Sub-County. 2019 [cited 12 Nov 2020]. Available: https://www.knbs.or.ke/?wpdmpro=2019-kenya-population-and-housing-census-volume-i-population-by-county-and-sub-county

2. Center For International Earth Science Information Network. Gridded Population of the World, Version 4 (GPWv4): Population Count, Revision 11. Palisades, NY: NASA Socioeconomic Data and Applications Center (SEDAC); 2018. doi:10.7927/H4JW8BX5

3. Knobel DL, Cleaveland S, Coleman PG, Fèvre EM, Meltzer MI, Miranda MEG, et al. Re-evaluating the burden of rabies in Africa and Asia. Bull World Health Organ. 2005;83: 360–368. doi:10.1590/S0042-96862005000500012

4. Morters MK, McKinley TJ, Restif O, Conlan AJK, Cleaveland S, Hampson K, et al. The demography of free-roaming dog populations and applications to disease and population control. J Appl Ecol. 2014;51: 1096–1106. doi:https://doi.org/10.1111/1365-2664.12279

5. Woodroffe R, Donnelly CA. Risk of contact between endangered African wild dogs Lycaon pictus and domestic dogs: opportunities for pathogen transmission. J Appl Ecol. 2011;48: 1345–1354. doi:https://doi.org/10.1111/j.1365-2664.2011.02059.x

6. Hampson K, Dushoff J, Cleaveland S, Haydon DT, Kaare M, Packer C, et al. Transmission Dynamics and Prospects for the Elimination of Canine Rabies. PLOS Biol. 2009;7: e1000053. doi:10.1371/journal.pbio.1000053

7. Stevenson M, Heuer C, Marshall J, Sanchez J, Thornton R, Reiczigel J, et al. epiR: Tools for the Analysis of Epidemiological Data. 2021. Available: https://CRAN.R-project.org/package=epiR

8. Spatial data and maps – Mpala. [cited 9 Mar 2021]. Available: https://mpala.org/data/spatial-data-and-maps/

9. Colombi D, Poletto C, Nakouné E, Bourhy H, Colizza V. Long-range movements coupled with heterogeneous incubation period sustain dog rabies at the national scale in Africa. PLoS Negl Trop Dis. 2020;14: e0008317. doi:10.1371/journal.pntd.0008317

10. Jandarov R, Haran M, Bjørnstad O, Grenfell B. Emulating a gravity model to infer the spatiotemporal dynamics of an infectious disease. J R Stat Soc Ser C Appl Stat. 2014;63: 423–444.

11. Li X, Tian H, Lai D, Zhang Z. Validation of the Gravity Model in Predicting the Global Spread of Influenza. Int J Environ Res Public Health. 2011;8: 3134–3143. doi:10.3390/ijerph8083134

12. Hampson K, Dushoff J, Bingham J, Brückner G, Ali YH, Dobson A. Synchronous cycles of domestic dog rabies in sub-Saharan Africa and the impact of control efforts. Proc Natl Acad Sci. 2007;104: 7717–7722. doi:10.1073/pnas.0609122104

13. Rajeev M, Metcalf CJE, Hampson K. Chapter 20: Modeling canine rabies virus transmission dynamics. In: Fooks A, Jackson A, editors. Rabies : Scientific Basis of the Disease and Its Management. 2020. pp. 655–670.
