# Supplementary material for: Modelling the influence of naturally acquired immunity from subclinical infection on outbreak dynamics and persistence of rabies in domestic dogs
Source: PLoS Negl Trop Dis. 2021 Jul 20;15(7):e0009581. doi: 10.1371/journal.pntd.0009581 (PMC8330898; doi:10.1371/journal.pntd.0009581)
Supplement: S2 Text — (PDF) [file pntd.0009581.s002.pdf]

S2 Text

Sensitivity analysis methods

A sensitivity analysis of the non-spatial model was conducted to explore the influence of uncertainty in the parameter estimates. In total 7 parameters were included (Maximum birth rate (a_max), background death rate (μ), probability of clinical infection (ϕ), probability of developing immunity(ρ), rate of loss of immunity (δ), rate of progression from exposed to infectious (σ) and rabies-induced death rate (ν)). The transmission rate (β) and q, the scaling parameter for relationship between birth rate and population size, were both calculated from other parameters and were therefore not included in the sensitivity analysis. R_0 and the carrying capacity (K) were kept constant. We selected 100 different parameter combinations using Latin hypercube sampling. Table A shows the limits between which each parameter was varied. For each parameter combination, 1000 simulations were run for 30 years. From each parameter combination we took the proportion of simulations in which rabies remained present, the total number of individuals infected and the seroprevalence in year 30. For these outputs we then computed partial rank correlation coefficients (PRCC) using epiR [1]. These coefficients provide an estimate of the strength of the relationship between the parameter and model output when the effects of other parameters are controlled for.

Table A- Range of parameter values used in sensitivity analysis. *Parameters varied in the sensitivity analysis are shown in green. Parameters calculated from other parameters are shown in orange. Fixed parameters are shown in yellow.*

Epidemiological description	Parameter	Range
**Basic reproduction number**	R_0	Kept constant at 1.2
**Maximum birth rate**	a_max	**0.0027-0.01** per dog per day
**Background death rate**	μ	**0.0009-0.0027** per dog per day (1-3 years average lifetime)
**Scaling parameter for relationship between introduction rate and population size**	q	Calculated from a_max and b
**Exposure rate per infectious individual per day**	β	Calculated from ϕ, ν and R_0
**Probability of developing clinical infection after exposure**	ϕ	**0.05-0.95**
**Probability of developing immunity following subclinical exposure**	ρ	**0-1**
**Rate of progression to clinical infection**	σ	**0.02-0.06** per exposed individual per day (16-50 days average duration)
**Rate of waning immunity**	δ	**0.00091- 0.01** per immune individual per day (100 days to 3 years average duration)
**Rate of rabies mortality**	ν	**0.14-1** per infectious individual per day (1-7 days average duration)
**Carrying capacity and starting population**	K	Kept constant at 63,434

Sensitivity analysis results

The sensitivity analysis showed that the probability of rabies persisting was sensitive to all parameters with the exception of the rate of progression from exposed to infectious (σ) and the rabies-induced death rate (ν). Decreasing the background death rate and increasing the birth rate were significantly associated with increased persistence (PRCC: 0.38, p<0.001 and PRCC: -0.21, p=0.04 respectively). The total number of individuals infected was also sensitive to these demographic parameters (a_max- PRCC: 0.61, p<0.001 and μ- PRCC:-0.21, p=0.04). This result suggests that dog demographics are important for rabies persistence, as if birth rates are lower or dogs die more rapidly the ability to replenish the susceptible population and therefore maintain rabies falls, and therefore less individuals in total are infected.

A lower proportion of individuals developing clinical infection (ϕ) and a higher proportion developing immunity (ρ) was significantly associated with increased probability of persistence (PRCC: -0.37, p<0.001 and PRCC: 0.22, p=0.027 respectively). Increasing the rate at which immunity wanes was also significantly associated with decreased probability of persistence (PRCC: -0.28, p=0.004). This result supports the findings presented in the main text that incorporating naturally acquired immunity increases the probability of persistence in the absence of spatial structure. In addition, as expected, seroprevalence was significantly sensitive to the parameters related to naturally acquired immunity ϕ (PRCC: -0.45, p<0.001) and ρ (PRCC: 0.36, p<0.001). However, the rate at which immunity wanes (δ) and the background death rate (μ) were not significantly associated with seroprevalence (PRCC: -0.16, p=0.09 and PRCC:-0.14, p=0.17).

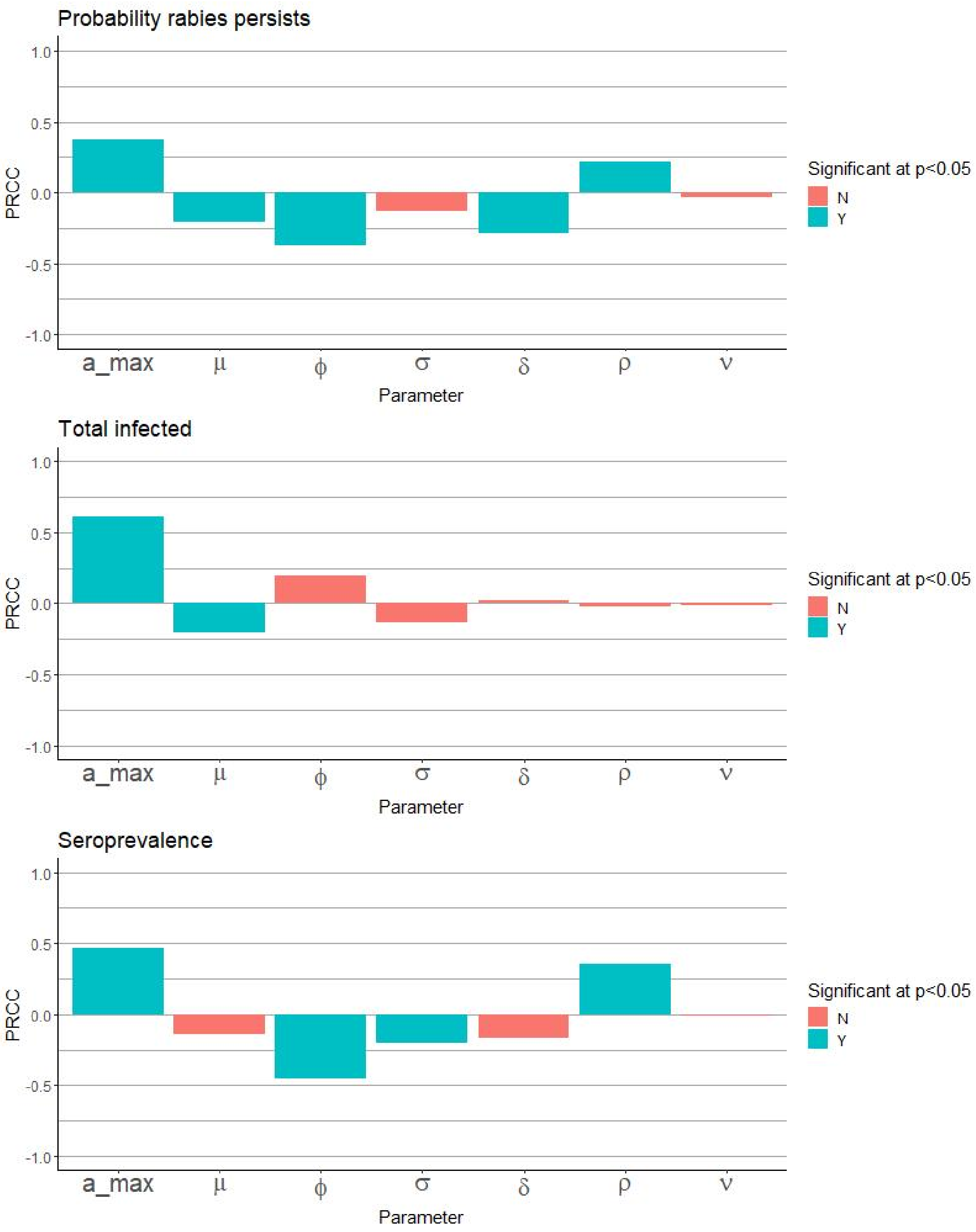

Fig A- Partial Rank Correlation Coefficients (PRCC) from sensitivity analysis. *Parameters included are birth rate (a_max), background death rate (μ), probability of developing clinical infection (ϕ), rate of progress to clinical infection following exposure (σ), rate of waning immunity (δ), probability of developing immunity (ρ) and rate of death from infection (ν). A positive PRCC value indicates that as the parameter is increased, the model output also increases (e.g. increasing persistence probability) whereas a negative value indicates as the parameter value increases, the model output decreases (e.g. decreasing persistence probability). Colour indicates whether the relationship is significant at the 0.05 level.*

References

1. Stevenson M, Heuer C, Marshall J, Sanchez J, Thornton R, Reiczigel J, et al. epiR: Tools for the Analysis of Epidemiological Data. 2021. Available: https://CRAN.R-project.org/package=epiR
