# Supplementary material for: Modelling the influence of naturally acquired immunity from subclinical infection on outbreak dynamics and persistence of rabies in domestic dogs
Source: PLoS Negl Trop Dis. 2021 Jul 20;15(7):e0009581. doi: 10.1371/journal.pntd.0009581 (PMC8330898; doi:10.1371/journal.pntd.0009581)
Supplement: S1 Table — (PDF) [file pntd.0009581.s003.pdf]

S1 Table- Median and interquartile ranges for all parameter combinations of ϕ and ρ for non-spatial model. *The medians from this table are presented in the heatmap in Fig 2 in the main text.*

Probability of clinical infection (ϕ)	Probability of immunity (ρ)	Median population decline (IQR)	Median annual incidence per 100,000 (IQR)	Median seroprevalence (IQR)	Proportion of endemic runs	Median persistence time in days (IQR)
0.05	0.00	1 (0)	0 (0)	0 (0)	0.00	1331 (161)
0.05	0.25	0.07 (0.01)	4843 (1384)	0.16 (0.04)	0.98	10949 (0)
0.05	0.50	0.04 (0.01)	2207 (1181)	0.15 (0.06)	0.87	10949 (0)
0.05	0.75	0.01 (0.02)	0 (1521)	0 (0.15)	0.46	10081 (6113)
0.05	1.00	0 (0.01)	0 (0)	0 (0)	0.18	4534 (6895)
0.28	0.00	1 (0)	0 (0)	0 (0)	0.00	1317 (147)
0.28	0.25	0.49 (0.02)	31325 (3678)	0.14 (0.02)	0.98	10949 (0)
0.28	0.50	0.26 (0.02)	16810 (2653)	0.15 (0.02)	0.98	10949 (0)
0.28	0.75	0.18 (0.02)	11445 (1982)	0.16 (0.02)	0.99	10949 (0)
0.28	1.00	0.13 (0.02)	8608 (1880)	0.16 (0.03)	0.99	10949 (0)
0.50	0.00	1 (0)	0 (0)	0 (0)	0.00	1317 (147)
0.50	0.25	0.96 (0.02)	61821 (19139)	0.11 (0.05)	0.94	10949 (0)
0.50	0.50	0.63 (0.03)	39679 (4841)	0.14 (0.02)	0.99	10949 (0)
0.50	0.75	0.44 (0.03)	28119 (3476)	0.15 (0.02)	0.98	10949 (0)
0.50	1.00	0.34 (0.02)	21631 (2923)	0.15 (0.02)	0.99	10949 (0)
0.73	0.00	1 (0)	0 (0)	0 (0)	0.00	1324 (161)
0.73	0.25	0 (0.01)	0 (0)	0 (0)	0.00	4152 (875)
0.73	0.50	0.73 (0.81)	0 (0)	0 (0)	0.18	8723 (2846)
0.73	0.75	0.93 (0.02)	59550 (11778)	0.12 (0.03)	0.99	10949 (0)
0.73	1.00	0.78 (0.03)	49308 (6497)	0.13 (0.02)	0.99	10949 (0)
0.95	0.00	1 (0)	0 (0)	0 (0)	0.00	1324 (154)
0.95	0.25	0 (0.01)	0 (0)	0 (0)	0.00	2332 (672)
0.95	0.50	0 (0.01)	0 (0)	0 (0)	0.00	2675 (441)
0.95	0.75	0 (0.01)	0 (0)	0 (0)	0.00	2885 (483)
0.95	1.00	0 (0.01)	0 (0)	0 (0)	0.00	3165 (602)
