# Supplementary material for: Modelling the influence of naturally acquired immunity from subclinical infection on outbreak dynamics and persistence of rabies in domestic dogs
Source: PLoS Negl Trop Dis. 2021 Jul 20;15(7):e0009581. doi: 10.1371/journal.pntd.0009581 (PMC8330898; doi:10.1371/journal.pntd.0009581)
Supplement: S1 Fig — (PDF) [file pntd.0009581.s004.pdf]

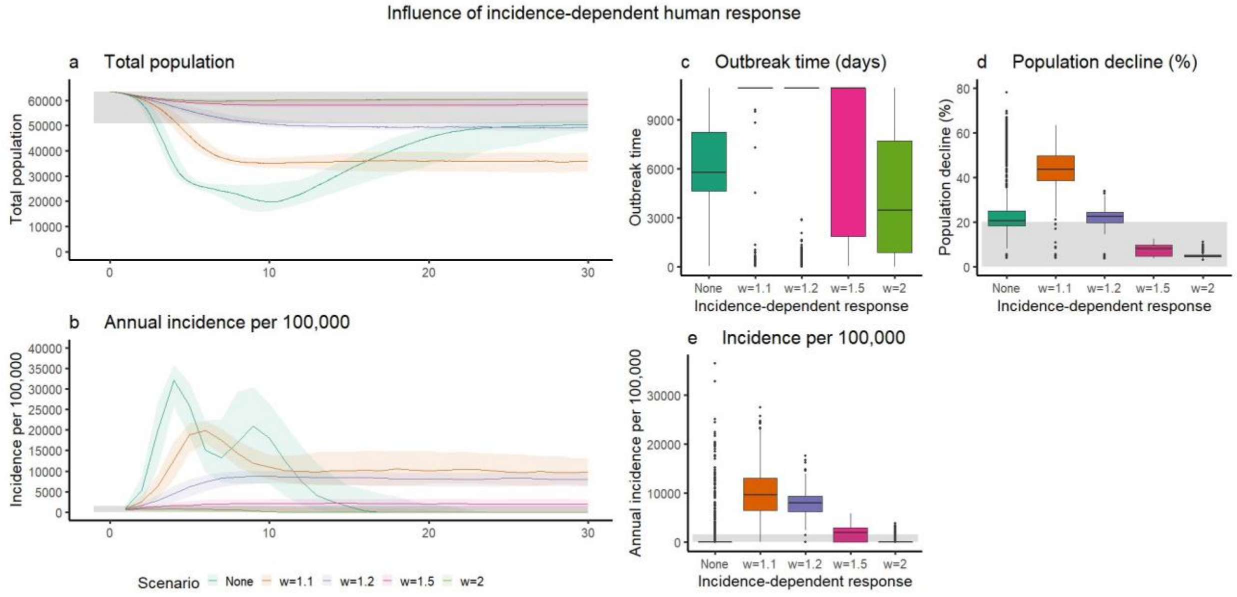

S1 Fig- Influence of incidence-dependent human response on model outputs in the absence of naturally acquired immunity (Scenario A). *When the monthly number of rabies cases within a patch is greater than a specified threshold of 1% of the patch carrying capacity, the mortality rate for infectious individuals (v) is multiplied by the w. Therefore w=2, represents a halving of the duration of the infectious period. Median and interquartile range for the total population size and annual incidence for each value of w over the 30-year simulation are shown in panels a and b respectively. Panels c-e in Fig B show boxplots for the outbreak time (c) (time from introduction to no infectious individuals remaining, or end of simulation), population decline (d) relative to the carrying capacity and incidence per 100,000 dogs in year 30 post introduction (e). Grey bands indicate the range of values considered plausible based on empirical estimates, as shown in Table 3 in the main text.*
