# Supplementary material for: Modelling the influence of naturally acquired immunity from subclinical infection on outbreak dynamics and persistence of rabies in domestic dogs
Source: PLoS Negl Trop Dis. 2021 Jul 20;15(7):e0009581. doi: 10.1371/journal.pntd.0009581 (PMC8330898; doi:10.1371/journal.pntd.0009581)
Supplement: S2 Fig — (PDF) [file pntd.0009581.s005.pdf]

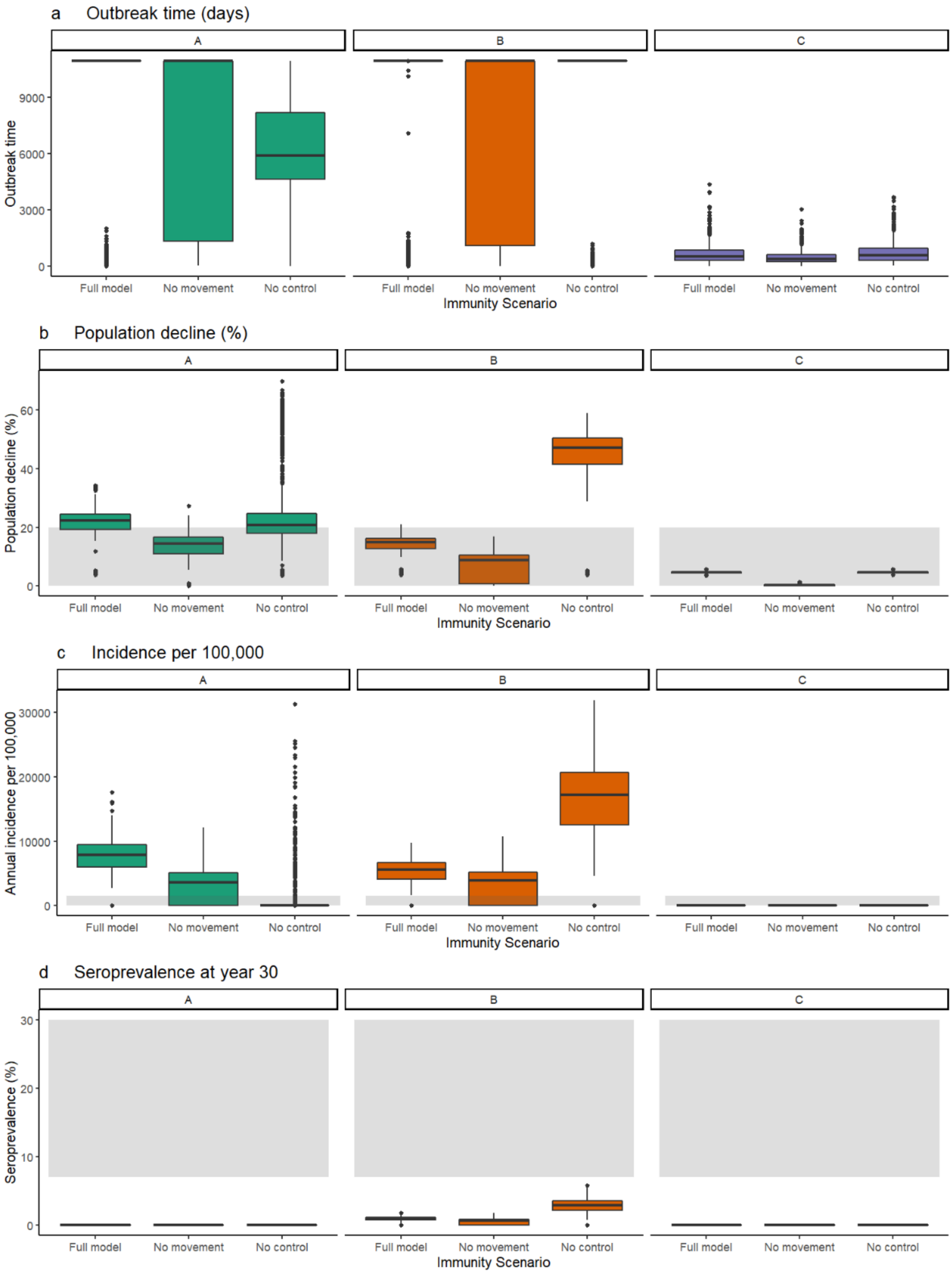

S2 Fig- Comparison of model outputs from immunity scenarios (A, B and C) across different model formulations for R_0 =1.2. *Results are shown from full model including both human mediated movement and incidence-dependent human response (Full model), and excluding human mediated movement (No movement) and incidence dependent human response (No control). Boxplots are shown for the outbreak time (a) (time from introduction to no infectious individuals remaining, or end of simulation), population decline (b) relative to the carrying capacity, incidence per 100,000 dogs in year 30 post introduction (c) and seroprevalence at the end of the simulation (d). Grey bands indicate the range of values considered plausible based on empirical estimates, as shown in Table 3 in the main text.*
